# Evolution of phosphate scouting in the terrestrial biosphere

**DOI:** 10.1098/rstb.2023.0355

**Published:** 2024-09-30

**Authors:** Steffen Abel, Christin Naumann

**Affiliations:** ^1^ Department of Molecular Signal Processing, Leibniz Institute of Plant Biochemistry, Halle 06120, Germany; ^2^ Institute of Biochemistry and Biotechnology, Martin-Luther-University Halle-Wittenberg, Halle 06120, Germany; ^3^ Department of Plant Sciences, University of California-Davis, Davis, CA 95616, USA

**Keywords:** biochemistry, geochemistry, evolution, phosphate acquisition, phosphate sensing, plant biology

## Abstract

Chemistry assigns phosphorus and its most oxidized form, inorganic phosphate, unique roles for propelling bioenergetics and metabolism in all domains of life, possibly since its very origin on prebiotic Earth. For plants, access to the vital mineral nutrient profoundly affects growth, development and vigour, thus constraining net primary productivity in natural ecosystems and crop production in modern agriculture. Unlike other major biogenic elements, the low abundance and uneven distribution of phosphate in Earth’s crust result from the peculiarities of phosphorus cosmochemistry and geochemistry. Here, we trace the chemical evolution of the element, the geochemical phosphorus cycle and its acceleration during Earth’s history until the present (Anthropocene) as well as during the evolution and rise of terrestrial plants. We highlight the chemical and biological processes of phosphate mobilization and acquisition, first evolved in bacteria, refined in fungi and algae and expanded into powerful phosphate-prospecting strategies during land plant colonization. Furthermore, we review the evolution of the genetic and molecular networks from bacteria to terrestrial plants, which monitor intracellular and extracellular phosphate availabilities and coordinate the appropriate responses and adjustments to fluctuating phosphate supply. Lastly, we discuss the modern global phosphorus cycle deranged by human activity and the challenges imposed ahead.

This article is part of the theme issue ‘Evolution and diversity of plant metabolism’.

## 1. Introduction

Phosphorus (P) is a biogenic element critical for all domains of life. Its sparse geochemical distribution and rare bioavailability determine marine primary productivity, often restrain terrestrial ecosystem growth and restrict biosphere size over geological timescales [1]. Uncommon chemical properties of the element are responsible for its exceptionality in the biosphere, which assigns P at its predominant pentavalent oxidation state on Earth, orthophosphate (PO_4_^3−^) or inorganic phosphate (Pi), a central place in biology, and empowers conjugate phosphate esters and phosphoric anhydrides to assume universal functions in cellular and organismal biochemistry [2,3]. The moderately strong, tribasic phosphoric acid dissociates its first proton at acidic pH, which renders the monoanionic (p*K*_*a*_ ~2) and dianionic (p*K*_*a*_ ~7) phosphate pair (H_2_PO_4_^−^/HPO_4_^2−^) an ideal biological buffer system at near-neutrality. Likewise, phosphate monoesters and diesters ionize readily at physiological pH, and the phosphate moiety is an excellent leaving group in metabolic nucleophilic substitution and elimination reactions. Phosphate esterification of many metabolites, including several coenzymes, is a common strategy to increase solubility, to chemically activate intermediates and to promote electrostatic attraction to enzymes or repulsion by lipid membranes, which confines phosphate esters within cells and subcellular compartments. In addition, the phosphate diester bridge is a recurring design principle in structural cell components such as phospholipids and nucleic acids. The remarkable inertia of the negatively charged phosphodiester linker to spontaneous water hydrolysis explains the extraordinary chemical stability of DNA (half-life >10^7^ years), which is essential for the faithful transmission of the genetic information encoded. More importantly, phosphoric anhydrides, such as nucleoside diphosphate or nucleoside triphosphate (NDP or NTP), are ideally suited to capture, conserve, distribute and donate free energy for facilitating unfavourable chemical reactions or biological processes. Due to its compact polyanionic nature, a single phosphoric anhydride moiety is under considerable electrostatic strain and thus thermodynamically unstable (storing a discrete portion of free energy, 20–40 kJ mol^−1^, for intracellular energy conversion); however, its P–O–P linkage is shielded by the phalanx of negative charges, averting nucleophilic attack and bestowing kinetic stability. Yet, powerful enzymes evolved to accelerate the specific transfer of phosphoryl groups by factors greater than 10^16^, which enable tight kinetic control over bioenergetics, metabolic flux, regulation of protein activity or replication and translation of nucleic acids [2–4].

De novo formation of phosphoric anhydride bonds in oxygenic photosynthesis, i.e. the light-driven, forced conjugation of Pi and ADP to ATP, constitutes the major chemical gateway for the portable entry of solar radiant energy into the biosphere. Thus, the availability of *phōs-phoros*, quite literally a *light-bearer* (Greek mythology) indeed, directly affects vital functions of photosynthetic organisms and boosts net primary productivity. A single P atom facilitates the assimilation of as many as 16 nitrogen (N) and 106 carbon (C) atoms into plant biomass, known as the Redfield ratio [5]. The current global biomass across all domains of life, approximated at 550 billion tonnes (Gt) C, is overwhelmingly dominated by plants in terrestrial (>450 Gt C) and much less in aquatic (<1 Gt C) habitats [6]. Estimates of the total amount of P in land plants vary between 0.5 and 3 Gt, which must be scavenged at an annual assimilation rate of 0.07 to 0.1 Gt P from the environment to meet metabolic demand, illustrating the enormous biogeochemical fluxes of the vital element in the biosphere [5,7]. This review highlights the evolution of plant Pi mobilization and acquisition strategies, ranging from the prebiotic Earth to the rise and dominance of terrestrial plants. First, however, we will trace the peculiarities of phosphorus astrochemistry and geochemistry.

## 2. Chemical evolution and geochemistry of phosphorus

Among the six major biogenic elements on Earth (H, C, O, N, S and P), the abundance of P is least favoured by stellar and planetary chemistry. Less than 10% of typical galactic stars are massive enough to explosively ignite precursor nuclei (C, O and Ne) in their hot cores, which give rise to third-row elements, including P [8]. Complex nucleosynthetic pathways lead to ^31^P, the single stable P nuclide, however, with only very low overall nuclear reaction yields (<2.5%). Thus, spurious P nucleosynthesis during the late evolution of massive stars followed by core-collapse supernovae likely explains the scarcity though the ubiquitous distribution of P in the universe. Its cosmic abundance (ranking 18th place) is by several orders of magnitude lower than that of the other five biogenic elements, rendering P the most critical chemical element for sustaining life in the universe from the perspective of astrobiology [1].

**Figure 1.**
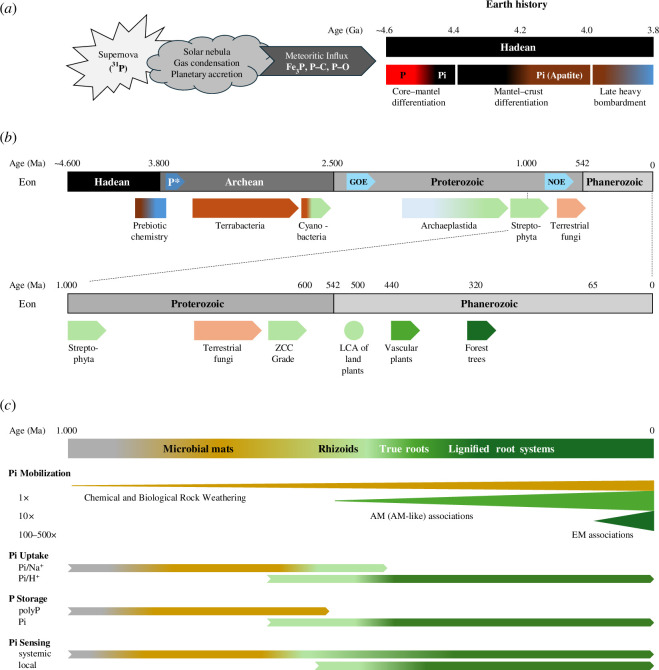
Evolution of Pi scouting from prebiotic Earth to the rise of terrestrial plants. (*a*) P cosmochemistry and Pi geochemistry on ancient Earth (Hadean aeon). Depicted are major processes of stellar P evolution and planetary P fractionation between the Earth’s liquid metallic core (red), harbouring reduced P (mainly as phosphide), and its mantel (black) and crust (brown), which host about 5% of the global P inventory as oxidized P (mostly Pi in apatite mineral phases). During the Late Heavy Bombardment, a disproportionately large number of asteroids and comets collided with Earth, possibly delivering vast amounts of water in ice and rock minerals that may have formed the prebiotic Archean Ocean (blue) [11]. (*b*) Evolutionary trajectories to terrestrial plants since the late Hadean aeon of Earth’s history. Approximate time spans of major evolutionary events on the path to land plants are indicated relative to the succession of geological aeons. Arrow-pointed boxes (blue) embedded within the timeline of Earth’s history mark the formation of reactive P at the end of the heavy bombardment (P*), the Great Oxidation Event (GOE) and the Neoproterozoic Oxidation Event (NOE). While the GOE transformed the anoxic atmosphere to a persistently oxic state with only a minute fraction of present atmospheric O_2_ levels, the NOE stepwise raised O_2_ to modern atmospheric concentrations [12,13]. The intervening period (1.8–0.8 Ga, occasionally called ‘the boring or barren billion’ [12,14]) experienced protracted tectonic, climatic and atmospheric stasis, which may explain the slow evolution of complex life despite the origin of the eukaryotic cell. Below the timeline (0–4600 Ma), arrow-pointed boxes (indicating continual evolution) mark the estimated first emergence of: Terrabacteria (brown); oxygenic photosynthesis in Cyanobacteria (brown to light green), which are one phylum of Terrabacteria; Archaeplastida (light blue to light green), which are photosynthetic eukaryotes including Glaucophyta, Rhodophyta and Chloroplastida; Streptophyta (light green), which evolved from Chloroplastida together with Chlorophyta; and terrestrial fungi (light brown). The lower timeline (0–1000 Ma) highlights the evolution of Streptophyta composed of streptophyte algae and Embryophyta (land plants). The paraphyletic ZCC grade (for Zygnematophyceae, Coleochaetophyceae and Charaophyceae; light green) of streptophyte algae arose from the paraphyletic KCM grade (for Klebsormidiophyceae, Chlorokybophyceae and Mesostigmatophyceae) at about 700 Ma. The Zygnematophyceae are the closest algal relatives to the hypothetical last common ancestor (LCA) of the monophyletic land plants (light green disc) [15]. The emergence of vascular plants and deep-rooted forest trees, which greatly intensified Pi acquisition, are indicated by green arrow-pointed boxes. (*c*) Evolution of Pi acquisition strategies. The timeline (0–1000 Ma) corresponds to panel (*b*) but highlights the role of microbial mats (coloured in ochre for the presence of photosynthetic and non-photosynthetic organisms) and of different rooting systems (coloured in green shades). The processes of chemical and biological Pi solubilization from rocks and of biochemical Pi re-mineralization from soil organic material (see §3) set the baseline for Pi mobilization in microbial mats (1×). Symbiotic associations of roots and possibly rhizoids with arbuscular mycorrhizal fungi and of tree roots with EM fungi amplify Pi mobilization rates by about tenfold and by more than 100-fold, respectively, which the thickness of coloured triangles alludes to. The evolutionary transitions of molecular Pi uptake systems (from Pi/Na^+^ to Pi/H^+^ co-transporters) and modes of Pi storage (from polyP polymers to free Pi) are depicted by the length (geological time) and colour (plant or non-plant organisms) of the arrows (see §6). Likewise, while the basic modules of systemic Pi sensing are evolutionarily conserved from fungi to plants (see figure 2), the mechanisms of local Pi sensing in roots arose during plant terrestrialization and partially relied on horizontal gene transfer (HGT) from Terrabacteria to the common ancestor of Streptophyta (see §6).

The vast majority of ^31^P ejected into interstellar space is effectively removed from the gas phase by elemental co-condensation with decreasing temperature. In early solar nebulae, the moderately volatile P condenses (<1200 K) onto nascent metallic Fe and Ni to form alloy phosphide dust, foremost (Fe,Ni)_3_P known as schreibersite, which oxidizes at a lower temperature (approx. 850 K) to orthophosphate minerals of the apatite group (table 1 in appendix A) [9]. Such microscopic grains, together with C condensates and diverse oxides of the rock-forming elements (mainly Si, Al, Fe, Mg, Ca, Na and K), constitute interstellar dust particles [8]. Coalescence of proto-planetary disc material and accretion of planetesimals into planetary bodies, which are large enough to experience melting, promote gravitational segregation of metals and associated siderophilic (metal-preferring) phosphides into planet cores, and of bulk silicates along with lithophilic (rock-preferring) phosphates to planet mantels. These processes likely explain the core–mantel differentiation of our planet at >4.4 billion years ago (Ga), followed by the heavy meteoritic bombardment period and mantel–crust differentiation during the Hadean aeon (4.4–4.0 Ga), which led to the large-scale fractionation of terrestrial P reservoirs [10]. Reduced P (mainly phosphide) is abundant in the liquid metallic core (3000 ppm), which contains approximately 95% of the global P inventory; however, the element is notably depleted from bulk silicate mantel rock (90 ppm). In contrast, the strongly lithophilic nature of oxidized P (mostly orthophosphate) favoured apatite phosphates to partition into mineral phases of the continental crust (650 ppm) where apatite minerals account for most of the crustal P reservoir (figure 1*a*). Compared to the other biogenic elements, P is of minute abundance in the lithosphere (mean content approx. 0.1%), ranking 11th place among the Earth crust’s chemical elements, and of note, P crustal geochemistry also differs strikingly. Under extant terrestrial conditions, P is rarely redox-active and does not partition into the gas phase (atmosphere). Furthermore, orthophosphate is generally poorly reactive and almost insoluble at near-neutral pH in the presence of common divalent metals such as Ca, Mg or Fe. Therefore, P geochemistry is dominated by rock–water interactions and not by liquid–gas phase transitions or redox changes, which determine the solubility and mobility of the other biogenic elements in the lithosphere and biosphere [10,16,17].

The exceptional importance of Pi and phosphorylated metabolites to bioenergetics and biochemistry suggests a vital role for P at the dawn of life, which is difficult to conceptualize when considering the chemical inertia of Pi and its obscured occurrence in rock (e.g. apatite minerals) as the only significant source of P accessible in Earth’s crust. The puzzling, so-called ‘phosphate problem’ refers to the thermodynamically unfavourable phosphorylation of organics in aqueous solution (a dehydration reaction producing water) and to the high concentrations of soluble Pi required as a reactant [16,18–20]. However, prebiotic chemistry on its path to the origin of life emerged at the end of the heavy meteoritic bombardment (4.0–3.8 Ga) [21,22], and it benefitted from the substantial deposition of simple organic C molecules, reactive N compounds and reactive P in the form of phosphide minerals onto the surface of early Earth, delivered by interplanetary dust particles, countless meteorites and numerous large impactors [10,16,23]. Schreibersite is the overwhelmingly dominating phosphide mineral of extraterrestrial sources and may have contributed 1–10% of crustal P during the Hadean aeon [24].

Interestingly, (Fe,Ni)_3_P readily corrodes in natural waters to oxidized P compounds of various redox states such as hypophosphite, phosphite, hypophosphate, Pi or pyrophosphate, which explains the very rare occurrence of schreibersite in the extant Earth crust [10]. Because phosphite in the presence of iron produces various polyphosphates (polyP; i.e. phosphoric anhydride polymers or ‘activated Pi’), the surface chemistry of phosphide minerals is capable of spontaneously generating phosphorylated organic molecules at low temperatures (<100°C) [25–28]. Alternative sources of reactive crustal P include the reduction of Pi to phosphite by frequent lightning strikes (forming fulgurite rocks) [29], active volcanism or more pervasively via the concurrent oxidation of Fe^2+^ at the temperatures (150–200°C) that develop during diagenesis (sedimentary rock formation) [30]. Further, Pasek *et al*. [31] proposed that hydrothermal transformation of ultramafic rocks to serpentine minerals (<300°C) promotes the reduction of Pi present in olivine-rich minerals (table 1 in appendix A). Because olivine is the dominant mineral of the upper mantel, the authors reasoned that rock serpentinization likely provided a significant source of phosphite on early Earth prior to the development of its abundant crust [31]. Subsequent mobilization of phosphite, which is about 1000-fold more soluble than Pi, may thus have enriched the prebiotic Archean ocean (>3.5 Ga) with reactive P to accelerate the early biochemical evolution [19,20,30] (figure 1*b*).

Pi-utilizing and phosphite- or phosphonate-metabolizing genes are present in diverse extant bacterial phyla and possibly arose before the divergence of monoderm (Gram-positive) and diderm (Gram-negative) bacteria (approx. 3.5 Ga) [32,33]. Most interestingly, recent studies showed that microbial AMP-dependent phosphite dehydrogenases couple the oxidation of phosphite to Pi with the reduction of NAD^+^ to NADH and the substrate-level phosphorylation of AMP to ADP [34]. Thus, a dissimilatory phosphite oxidation pathway operates to generate the assimilatory power (ATP and NADPH) required for CO_2_ fixation that possibly proceeds via acetyl-CoA (‘activated acetic acid’) formation, the phylogenetically oldest reactions for autotrophic cell matter production known as the Wood–Ljungdahl pathway. Such reactions in extant bacteria may represent relics of ancient biochemistry and perhaps resolve the ‘phosphate problem’ at the dawn of life [34–36] (figure 1*b*).

## 3. Microbial acceleration of the global phosphorus cycle

### (a) Geochemical Pi cycling

About 95% of the lithosphere (Earth’s crust and upper mantel) is composed of silicate minerals (SiO_4_^4−^) hosting various metal cations (typically Al^3+^, Ca^2+^, Mg^2+^, Fe^2+/3+^ and others as present in granite or basalt rock). The most important P-bearing phases in the crust are minerals of the apatite group and to a lesser extent the heterogenous P-containing zones in mafic silicate minerals of the olivine group present in common igneous rock [10] (table 1 in appendix A). Apatite phases occur as early formed accessory minerals in felsic igneous or metamorphic rock (up to 1% fluorapatite and chlorapatite) and as chemical precipitates in sedimentary rock (up to 80% authigenic hydroxy- and carbonate-fluorapatite). The atmospheric CO_2_ concentration and global surface temperature significantly control the chemical stability of apatite, which both have dramatically fluctuated across a wide range of geochemical conditions during the Phanerozoic aeon (past 542 Myr) [10,37]. Geological erosion and chemical weathering of silicate rock via acidic attack (rainwater-dissolved HCO_3_^−^) solubilizes various metal ions and Pi from apatite and olivine minerals. Congruent igneous rock weathering sequesters Pi and metal cations into salt precipitates or carbonate-Pi co-precipitates. Similarly, strong adsorption of Pi onto the large surfaces of abundant metal oxides and oxyhydroxides forms recalcitrant mineral phases (mainly Fe and Al) or secondary clay minerals (foremost Si), which often constitute the main pool of environmental Pi. Fluvial and to a lesser extent aeolian transport routes deliver the physically eroded and chemically weathered continental Pi in dissolved but mostly particulate form to the oceans, where it is buried on continental shelves and in deep seas. Riverine Pi, foremost as particulate matter, presents the single largest influx of continental P to the oceans. Sediment accretion and subduction, melting of sedimentary rock in the upper mantle, followed by igneous rock formation during volcanism or geotectonic uplift (e.g. the recent rise of the Himalayan plateau 10 Ma), complete the geochemical P cycle, which intersects with the global inorganic C (CO_2_-carbonate-silicate) cycle [38–40].

### (b) Microbial Pi solubilization

Physical and chemical weathering on the small specific surface area of pristine rock produces only thin layers of mineral dust and debris (regolith), but not true soil. The colonization of continents by bacteria occurred in the mid-Archean aeon (3.5–2.8 Ga) with the divergence of Hydrobacteria and Terrabacteria (figure 1*b*). Adaptations to terrestrial desiccation and harsh UV radiation accelerated prokaryote diversification, whereas rock weathering and leaching into oceans progressively deprived early prokaryotic colonists (anoxygenic phototrophs) of readily available mineral electron donors (mainly reduced iron and sulfur species), which imposed strong selection pressures for evolving oxygenic photosynthesis by cyanobacteria in moist land environments [12,41]. The use of water as an abundant electron donor and the simultaneous production of O_2_ as a diffusible electron acceptor in aerobic metabolism immensely increased net primary productivity compared to anoxygenic photosynthesis, gave rise to a UV-protective stratospheric ozone layer and promoted the evolution of multi-cellularity since the Great Oxidation Event (2.4–2.3 Ga) [13]. Microfossil and geochemical records point to the formation of biotic crusts and subaerial microbial mats by approximately 850 Ma, which likely hosted archaebacteria, photosynthetic bacteria (foremost cyanobacteria) and various unicellular and multi-cellular protists such as fungi and algae (figure 1*b*). The expansion of oxygenic photosynthesis in microbial terrestrial ecosystems likely contributed to the second stepwise rise of atmospheric O_2_ level since the onset of the Neoproterozoic Oxidation Event (800–600 Ma) [12]. The photosynthetically active ground cover significantly increased chemical rock weathering, mineral solubilization, first soil formation and mineral nutrient transport to the oceans [12,42,43]. Together with global abiotic erosion, such biotic processes contributed to the long-term geological transformation of Earth’s crust from broadly igneous bedrock to mainly sedimentary rock across the Neoproterozoic–Phanerozoic boundary (600–400 Ma) [40].

Nearly all accessible Pi on freshly exposed rock surfaces is locked in apatite phases. Microorganisms amplify the chemical and, indirectly, the physical weathering of Pi-hosting minerals, commonly referred to as biological weathering. One prominent process for microbially mediated leaching of soluble Pi from rock includes the local elevation of humidity and acidity in the substratum, which is often intensified by biofilm aggregation. Numerous microbial taxa release protons (e.g. ATPase-mediated proton translocation) and organic acids, and chemoautotrophic bacteria produce inorganic acids such as nitric or sulfuric acid by oxidizing ammonia or sulfur, respectively. In addition to biogenic acid production, which is augmented by respiratory CO_2_ release and carbonic acid formation in moist surroundings, various bacterial redox reactions solubilize Pi via the reduction of iron oxyhydroxide and associated ferric phosphate (FePO_4_). For example, dissimilatory Fe^3+^ reduction generates soluble Pi and Fe^2+^, and S-reducing bacteria produce H_2_S, which reduces FePO_4_ to soluble Pi and iron sulfide precipitates (pyrite). Similar mineral mining strategies, which concurrently increase Fe and Pi bioavailability, involve the release of soluble organic electron shuttles (redox-active metabolites) and the extrusion of organic chelating compounds (e.g. siderophores, phenolics or carboxylates such as citrate, oxalate or malate), which reversibly complex metal cations [39,44–46].

### (c) Microbial Pi re-mineralization

In addition to Pi solubilization from rock, microorganisms scavenge Pi by enzymatic attack of otherwise inaccessible organic P-containing compounds present in decaying dead biomass of the growth substrate, processes that generate and recycle free Pi for all trophic levels [1,39]. A suite of secreted phosphohydrolases acts on nucleic acids, phospholipids, numerous phosphate esters and various types of phosphoric anhydrides such as nucleoside triphosphates (NTPs), pyrophosphate or polyP to liberate Pi for subsequent uptake by various Pi transporters, a process termed Pi re-mineralization. Many microorganisms digest external DNA and RNA polymers as an important organic source of nutrients and mineralize Pi in two stages. Bifunctional nucleases and monofunctional ribonucleases or deoxyribonucleases depolymerize nucleic acids, and nucleotidases hydrolyze the constituent nucleoside monophosphates (NMPs) into nucleosides and soluble Pi. Additional enzymes dedicated to Pi re-mineralization comprise the diverse class of phosphomonoesterases with a broad substrate spectrum of phosphorylated compounds, substrate specificities and affinities, pH optima (acid or alkaline phosphatases) and other biochemical properties for optimal catalytic activity. Notable examples of phosphatases are secreted phytases hydrolyzing *myo*-inositol 1,2,3,4,5,6-hexakisphosphate (phytate or IP_6_), which strongly sorbs onto soil particles and often represents a significant fraction of extracellular organic P [47].

Terrestrial and especially marine bacteria can utilize and oxidize inorganic phosphite and derived organic phosphonates as a potential source of Pi [1,48,49]. While the assimilatory phosphite oxidation pathway channels the generated Pi into anabolic metabolism, the dissimilatory pathway uses phosphite as the sole electron donor and source of chemical energy, coupling phosphite oxidation to ATP and NADH production, which is possibly followed by Pi uptake and Pi assimilation [32,33]. More than one-third of bacteria can metabolize a wide range of organic phosphonate compounds via a C–P lyase multi-enzyme complex of broad substrate specificity, which cleaves the characteristic but chemically stable C–P bond to form Pi and hydrocarbons [48].

As a result, microbial Pi solubilization and re-mineralization may locally increase the concentration of free Pi in terrestrial and aquatic habitats. Pi supersaturation of the wet milieu causes the formation of insoluble metal–Pi co-precipitates and of stable minerals such as apatite or, as found in marine sediments, phosphorites that contain significant amounts of various Pi-containing minerals [39]. The microbial processes dedicated to Pi foraging and acquisition are under genetic control, and the expression of the relevant proteins and enzymes is typically activated in conditions of low external Pi availability.

## 4. Genetic control of phosphate scouting by extant microbiota

### (a) Bacterial Pho regulation

In prokaryotes, a widely conserved regulatory gene network, the Pho regulon, governs external Pi mobilization and acquisition, and it maintains internal Pi homeostasis by recalibrating metabolic Pi allocation. First characterized in *Escherichia coli* and later described in many bacterial phyla, including Actinobacteria and photosynthetic Cyanobacteria, the bacterial Pho regulon comprises several operons with over 30 Pho genes, which encode various phosphohydrolases, Pi and phosphite import systems of high and low affinities, enzymes of phosphonate metabolism and proteins dedicated to reversible linear Pi polymerization and storage of osmotically inactive polyP in acidocalcisome-like granules [47,48,50–52]. In all domains of life, cellular functions of polyP range from the storage of Pi and metabolic energy (phosphoric anhydride bonds) to protective roles as antioxidants (sequestration of metals) or chaperones that alleviate stress-induced protein aggregation [53,54].

**Figure 2.**
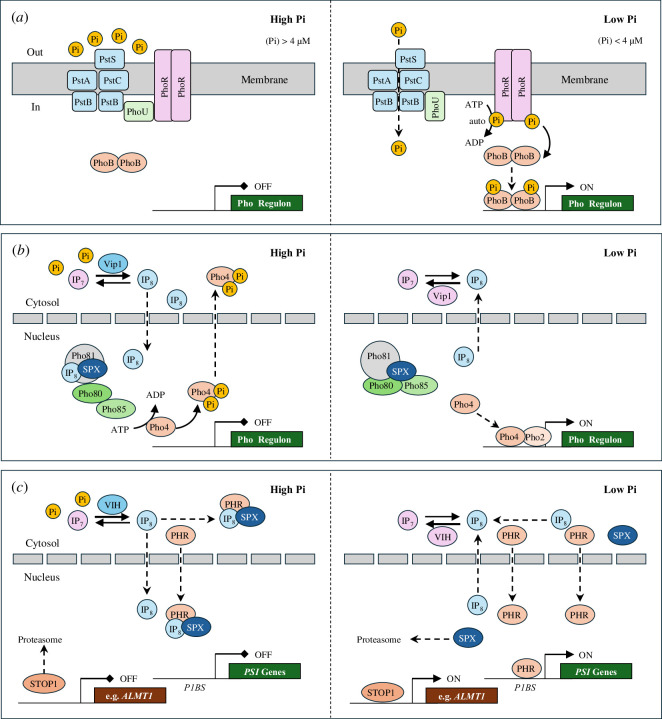
Minimal models of Pi sensing and core transcriptional response circuits. (*a*) Pho regulation in bacteria (*E. coli*). The Pi-specific transporter (Pst), composed of four different Pst subunits (blue), functions as a sensor of extracellular Pi. In Pi sufficiency, Pi binds to PstS and inactivates PhoR, a histidine sensor kinase, via PhoU-mediated recruitment to PstB. In Pi limitation, Pi is taken up by Pst, which activates autophosphorylation of PhoR, subsequent phosphorylation and activation of PhoB, a transcriptional response regulator of diverse Pho box-containing promoters (Pho regulon). (*b*) Pho regulation in yeast (*S. cerevisiae*). In Pi sufficiency, Vip1, a bifunctional IP_7_ kinase/IP_8_ phosphatase signals intracellular Pi status via the dynamics of IP_8_ level to the cell nucleus. IP_8_ binds to the SPX domain of Pho81, an inhibitor of the cyclin-dependent protein kinase complex, Pho85 (kinase)–Pho80 (cyclin), which causes partial Pho81 dissociation and thus Pho85–Pho80 kinase activation. Phosphorylation of Pho4, a basic helix-loop-helix transcription factor facilitates nuclear Pho4 export and Pho gene repression. In Pi limitation, IP_8_ level drops (activation of Vip1 IP_8_ phosphatase), which represses Pho85–Pho80 kinase activity via tighter binding to Pho81. Dephosphorylated, nuclear Pho4 interacts with Pho2, a homeodomain transcription factor, to activate Pho gene expression. (*c*) Regulation of *PSI* genes in land plants (*A. thaliana*). Like in yeast, the dynamics of IP_7_/IP_8_ levels, responding to intracellular Pi and energy status via the bifunctional kinase/ phosphatase VIH, control the activation of Pi-responsive genes. In high Pi, IP_8_ mediates the recruitment of SPX proteins to PHR transcription factors of the plant-specific MYB-CC class to repress *PSI* gene activation. When IP_8_ levels drop in low Pi conditions, PHR–SPX complexes dissociate, leading to *PSI* gene activation and SPX protein degradation. Unlike the systemic Pi response, local Pi sensing of extracellular Pi availability is controlled by yet unknown processes (involving proteolysis), STOP1, a zinc finger transcription factor, which activates some of its target genes in low Pi, such as *ALMT1*. For details, see §§4 and 6 or recent reviews [50,55,56].

In *E. coli*, the hetero-oligomeric high-affinity ABC (ATP-binding cassette) Pi transporter complex, Pst (phosphate-specific), is the most conserved member among bacterial Pho regulons and catalyzes the uptake of Pi from the surrounding medium at low Pi concentrations (<4 µM), supplementing the activity of the constitutive low-affinity Pi transporter, Pit (phosphate inorganic transporter), at higher Pi availability. A two-component regulatory system, consisting of a transmembrane signalling histidine kinase sensor, PhoR, and a cytoplasmic transcriptional response regulator, PhoB, controls the expression of bacterial Pho regulons. Extracellular Pi availability is monitored by PhoR–PhoB in a complex with Pst and a modulatory subunit, PhoU. Under Pi deficiency, PhoR undergoes autophosphorylation followed by phosphoryl group transfer to PhoB, which recognizes Pho box consensus sequences of Pho promoters, leading to the initiation of Pho gene transcription. Under Pi sufficiency, PhoU together with the Pst negatively regulates PhoR–PhoB by preventing PhoR autophosphorylation, which promotes PhoB dephosphorylation and thus repression of Pho gene activation [47,50] (figure 2*a*).

### (b) PHO regulation in fungi

In fungi, best studied in budding yeast (*Saccharomyces cerevisiae*), a unicellular non-photosynthetic eukaryotic model, the PHO regulon (or PHO pathway) comprises numerous genes that are directly implicated in the maintenance of cellular Pi homeostasis [55,57,58]. While fungal Pi scavenging and recycling strategies, including many encoded PHO enzymes, are analogous to those in bacteria, the mechanisms of Pi sensing and signalling differ significantly between the two kingdoms. The PHO regulon in yeast primarily monitors intracellular Pi levels and mounts a fine-tuned response to fluctuating cellular Pi supply and metabolic demand (figure 2*b*). The PHO pathway is controlled by a Pi-responsive, nuclear-localized protein kinase complex (Pho85–Pho80–Pho81), which, in Pi sufficiency, phosphorylates the basic helix-loop-helix transcription factor Pho4 at multiple sites. Nuclear export of phosphorylated, inactive Pho4 protein abrogates PHO gene transcription. Aggravating Pi depletion increasingly promotes de-phosphorylation of Pho4, which stimulates its nuclear accumulation and association with the homeodomain transcription factor Pho2. Depending on the gravity of cellular Pi shortage, the Pho4–Pho2 heterodimer recruits the general transcription machinery to PHO promoters and gradually co-activates the expression of PHO gene subsets with partially overlapping Pi dose responses. The proteins encoded are essential for growth and survival in Pi-limiting environments and function in Pi uptake and storage, external Pi scavenging and internal Pi recycling. Noteworthy is the differential expression and regulation of high-affinity (Pho84 and Pho89) and low-affinity (Pho87 and Pho90) Pi transporters, which also differ in pH optima and the ion gradient used (H^+^ or Na^+^) to power symport Pi uptake [55].

In yeast, the Pi-responsive nuclear protein complex consists of a cyclin-dependent protein kinase (CDK) subunit, Pho85, which associates with cyclin Pho80, one of at least ten different cyclins that integrate the relevant responses for maintaining cellular Pi homeostasis. A CDK inhibitor, Pho81, physically interacts and regulates Pho85–Pho80 kinase activity and the PHO pathway. Pho81 features an N-terminal SPX (Syg1/Pho81/XPR1) domain, which is frequently found in proteins with diverse roles for maintaining Pi homeostasis. SPX domains are widely conserved in eukaryotes and share positively charged surface clusters of conserved arginine and lysine residues, which bind pyrophosphate group-bearing *myo*-inositol phosphates (inositol pyrophosphates) with high affinity [59–61]. Cytosolic Pi sufficiency leads to an increased formation of *myo*-inositol 1,5-bis(diphosphate) 2,3,4,6-tetrakisphosphate (1,5-IP_8_ for short) at low steady-state level (approx. 0.3 µM [62]), which binds to the SPX domain of Pho81 and activates the Pho85–Pho80 kinase complex via partial Pho81 dissociation. In Pi depletion, declining 1,5-IP_8_ level restores the inhibitory association of ligand-free Pho81 with the Pho85–Pho80 complex, preventing Pho4 phosphorylation and activating PHO gene expression [62]. Vip1, a bifunctional 5-IP_7_ kinase/1,5-IP_8_ phosphatase of the diphosphoinositol pentakisphosphate kinase (PIPP5K) family, monitors and signals the intracellular Pi status via the dynamics of 1,5-IP_8_ level to Pho81 and possibly other proteins harbouring an N-terminal SPX sensor domain (figure 2*b*). Such proteins include Pi importers (Pho87 or Pho90), subunits of the VTC (vacuolar transporter chaperone) complex (Vtc1–Vtc5), which synthesizes and concurrently translocates polyP into the vacuole, or Pi exporters such as Pho91, a vacuolar permease containing an SPX and SCL (solute carrier) domain that releases Pi produced by vacuolar endo-polyphosphatase activity to the cytoplasm [63,64]. Notably, the 1,5-IP_8_ phosphatase activities of human PIPP5Ks are strongly inhibited by Pi, whereas the 5-IP_7_ kinase activities are reciprocally enhanced by Pi and increasing ATP [65]. Thus, the activity of Vip1 may transmit concentration changes of key energy metabolites (Pi and ATP) to SPX sensor domain-containing target proteins via synchronized, dynamic concentration changes of the 1,5-IP_8_ signalling molecule, a pathway termed INPHORS (intracellular Pi reception and signalling) [55,62].

### (c) Pho regulation in microalgae

The first photosynthetic eukaryotes probably emerged 1.9–1.2 Ga by the incorporation of a basal cyanobacterium within a heterotrophic eukaryote host. Primary endosymbionts (Archaeplastida) gave rise to the lineages of rhodophytes (red algae), glaucophytes and Chloroplastida. The latter, or so-called green lineage, evolved to the chlorophytes (green algae) and to the streptophytes, which separated into freshwater streptophyte algae and embryophytes (land plants) [15,66] (figure 1*b*). A myriad of phosphate starvation inducible (PSI) genes and proteins acclimatize *Chlamydomonas reinhardtii*, a well-studied model organism for unicellular green algae, to fluctuating Pi supply in aqueous or moist habitats [67]. Such *PSI* genes, which have been described in other marine microalgae, encode homologs of the yeast Pho84 and Pho89 high-affinity Pi transporters, homologs of yeast enzymes for polyP synthesis, storage in and mobilization from specialized acidocalcisome-like vacuoles, a wide spectrum of phosphatases and nucleases for Pi scavenging, enzymes for replacing membrane phospholipids with sulfolipids and galactolipids or proteins with functions in photosynthesis and general metabolism [68–72]. The expression of many *PSI* genes in algae is controlled by phosphorus starvation response 1 (PSR1) or PSR1-like members of the diverse myeloblastosis (Myb) family of nuclear transcription factors that are widespread in eukaryotes [71–73]. PSR1 features an MYB-like DNA-binding domain for activating *PSI* gene promoters and a coiled-coil (CC) domain for protein–protein interaction (MYB-CC) [69]. The presence of putative SPX proteins and homologs of the yeast SPX domain-containing subunit VTC4 in microalgae across three phyla point to conserved mechanisms of intracellular Pi sensing via inositol pyrophosphate signalling molecules to SPX receptor domains (see §6c) [71,74,75].

## 5. Amplification of global phosphate cycling by terrestrial plants

The evolution and rise of land plants in terrains initially dominated by microbial crusts significantly amplified global biogeochemical cycles. Plant-intensified biological weathering of igneous silicate and sedimentary carbonate rock accelerated flux of apatite-derived Pi on continents and to the oceans and nutrient cycling within terrestrial ecosystems via biochemical Pi re-mineralization, which increased net primary productivity, organic C burial and atmospheric O_2_ level since the Neoproterozoic Oxidation Event [76,77]. The common multi-cellular photosynthetic ancestor of the streptophytes acquired the ability to colonize subaerial and terrestrial habitats about 600 Ma. Phylogenetic analyses and biogeochemical modelling suggest that ancestral land plants emerged in the late Cambrian/early Ordovician period (510–490 Ma), after diverging from the Zygnematophyceae, the most closely related sister clade of freshwater and terrestrial streptophyte algae [37,76–79]. By the early Silurian period (approx. 440 Ma), the archaic embryophytes split into the bryophyte and tracheophyte lineages whose spore-producing members formed cryptogamic ground covers within the microbial mats. Vascular land plants were widespread by 370 Ma (mid-Devonian), and since the late Carboniferous period (approx. 320 Ma) arborescent forests dominated the continents [37,76–79] (figure 1*b*).

### (a) Mutualistic organismic interactions

The evolutionary transition of the land plant ancestor from aqueous to largely atmospheric exposure, leaving behind the benefit of immersion in water as a solvent for nutrients and protectant against excess (UV) light and high temperature, faced multiple obstacles and required the innovation of structural, physiological and biochemical adaptive strategies for survival in desiccating environments [80]. Water and mineral nutrient scarcity in the early nascent soils posed a major challenge to the first land plants with only simple rhizoid-based absorbing systems (terminal outgrowth of single filamentous cells). Therefore, the development of nutritionally mutualistic interactions with bacteria and fungi was one such key innovation [81,82].

More than 80% of the extant vascular plant species recruit arbuscular mycorrhizal fungi (AMF) of the Glomeromycota, which develop arbuscular structures in the cortex cell layers of roots and extensive hyphal networks in soil, for increasing drought tolerance and nutrient uptake (foremost Pi) in exchange for delivering carbohydrates and lipids [83]. Interestingly, Glomeromycota also establishes AM-like associations with rootless, non-vascular spore-forming plants [84], and the Mucoromycota, considered a more basal or sister fungal lineage, engage in comparable, AM-like mutualistic relationships with thalloid liverworts of the earliest diverging extant land plants (e.g. *Haplomitrium*). Thus, the coevolution of embryophytes and beneficial fungi to form mutualistic associations is thought to have facilitated early plant terrestrialization [81,85,86] (figure 1*c*).

### (b) Evolution of rooting systems

The algal ancestor of embryophytes was partially pre-adapted for fungal symbiosis, as suggested by the presence of key symbiotic signalling genes but the absence of downstream genes required for root colonization [87]. Algal sister lineages of land plants had already acquired the capacity to protrude primordial rhizoids for adhesion [15,88,89]. Formation of rhizoids in bryophytes, attaching the thallus to soil for mineral nutrient scavenging, suggests that rhizoid-based rooting systems represent the earliest anchorage strategy of land plants. Vascular plants of the late Silurian (approx. 420 Ma) lacked true roots, however, developed extended rhizomes, i.e. shallow rhizomatous axes furnished with unicellular root hairs, on or just beneath the soil surface [37,90]. Noteworthy, the molecular mechanisms controlling the development of rhizoids in bryophytes and of root hairs in tracheophytes are evolutionarily conserved [91,92].

True roots are a derived characteristic of vascular plants and evolved convergently at least twice in the lycophytes and euphyllophytes. Deep root systems were essential to the rise of arborescent trees and forested ecosystems. Almost all extant vascular plants develop a highly branched root system architecture, powered by numerous lateral meristems, for deep penetration and broad exploration of soil and bedrock resources [37,79]. While plant terrestrialization coincided with the rise of AMF, ectomycorrhizal (EM) fungi (Basidiomycota and Ascomycota) evolved independently much later (approx. 65 Ma) and colonize, often together with AMF, both gymnosperm and angiosperm tree roots to form extensive hyphal networks with large absorptive surface area [93,94] (figure 1*c*).

### (c) Pi mobilization by terrestrial plants

Embryophytes and their symbionts greatly amplify rock weathering and Pi extraction by several mechanisms. Development of a vast lignified root system in trees can physically fracture soil-covered bedrock and increase its specific surface area for chemical weathering and mineral nutrient extraction, expedited by fungal hyphae or lichens via the processes described above (see §3). Lichens are symbiotic organisms of chlorophyte algae and Ascomycota fungi, which postdate the emergence of embryophytes [80]. Evapotranspiration by the continental vegetation cover accelerates the global hydrological cycle, amplifying regional rainfall, dissolution of silicate minerals by carbonic acid and formation of secondary clay minerals. Root respiration and aerobic decay of organic soil detritus by microbial respiration further promote HCO_3_^−^ generation and soil acidification [37,76,95]. Plants deposit a considerable fraction of their net assimilated carbon into the rhizosphere, which may range from less than 10% to more than 40%, depending on plant species, developmental stage and nutrient status [96,97]. Functional root exudates mobilize Pi and other nutrients directly by promoting silicate weathering (organic acids) and metal chelation (phenolics and siderophores) and also indirectly by supporting large communities of root-associated microorganisms (plant growth-promoting rhizosphere bacteria and fungi) to establish mutualistic interactions [80,81,86].

Ultimately, the evolution of seed plants (gymnosperm and angiosperm) unleashed the expansion of vegetation cover from wet lowlands to dry upland habitats and stimulated the invasive spread of arborescent trees. Forested ecosystems with their extensive, often dual hyphal networks for aggressive chemical weathering (EM fungi) and effective Pi acquisition (AMF) have been likened to ‘major engines of continental silicate weathering’ [98]. While root hairs protrude a few millimetres into the rhizosphere, the hyphal network of AMF explores the soil several centimetres beyond the root, which is dramatically expanded to a few metres by EM fungi. Compared to plant-free controls, lichens and bryophytes with their associated mycorrhizal symbionts enhance the chemical weathering rate of Pi from granite or basalt grains about tenfold, which arborescent trees further amplify by a second order of magnitude (about 100-fold) [37,98,99] (figure 1*c*). Thus, the various powerful rooting strategies effectively scout and mobilize Pi from bedrock for uptake by plants and for reflux to soil from decaying plants (organic P and immobilized Pi in soil litter and detritus).

Importantly, a thriving plant cover promotes soil stability and obstructs soil erosion, particularly in mountainous ecosystems and sloping landscapes that dominate the terrestrial surface [100]. Reducing soil erosion by vegetation prolongs and intensifies the weathering of rocky soils (Pi solubilization) and multiplies the recycling of the nutrients within soil environments (Pi re-mineralization) [95]. Compared to models of global C and N cycles, the internal fluxes of Pi cycling between vegetation and soil dominate the global P cycle and are considerably larger than the external fluxes of Pi inputs (e.g. rock weathering) and Pi outputs (e.g. riverine transport to oceans) [7]. Obviously, the impact of land plant evolution on global Pi mobilization, here abridged as distinct developmental innovations and separate processes for enhancing terrestrial Pi acquisition, had profound complex consequences over geological timescales for Earth’s biosphere and climate via the interrelated and mutually feedback-controlled biogeochemical P, C and O_2_ cycles [37,76,77,95], a discussion of which however is beyond the scope of this review.

## 6. Evolution of phosphate scouting by extant land plants

While riverine transport of weathered Pi into the oceans limits marine primary production, the age of soil largely determines Pi availability and thus terrestrial net productivity. Soil development (pedogenesis), as studied by the succession of well-dated chronosequences (i.e. spatial gradients in geologic soil age), involves long-term depletion of exposed primary Pi minerals in bedrock or lava, continual formation of occluded secondary Pi minerals, biochemical transformation to P-containing organic matter and substantial loss of soluble and particulate Pi to surface waters [101,102]. The soil resources of P and N greatly co-limit plant growth and are strongly dependent on the stage of pedogenesis. N is absent in parental rock and enters ecosystems mainly via biological N_2_ fixation or atmospheric deposition. Therefore, primary productivity is low on young, P-rich soils (>4000 ppm P), increases with rising N_2_ fixation during pedogenesis until a plateau is reached (N/P co-limitation) and declines on old, highly weathered soils (<15 ppm P), leading to ecosystem retrogression. In addition, soil chemistry aggravates low Pi bioavailability during pedogenesis. Pi firmly adsorbs onto the large specific and occluded surface areas of metal (Fe and Al) oxides or hydroxides and secondary clay (Si) minerals. Most Pi salts, with a few exceptions (alkali metals), are notoriously insoluble and readily precipitate in the presence of metal cations in acidic (Al^3+^ and Fe^2+/3+^) or alkaline (Ca^2+^ and Mg^2+^) conditions. These properties severely restrict Pi mobility in soil (diffusion coefficient <10^−12^ m^2^ s^−1^) and Pi concentration in soil solution (<10 µM) [103–106].

### (a) Plant Pi acquisition strategies

In contrast to the high mobility of nitrate in soil, the slow Pi mass flow contrasts with the high Pi uptake rate by roots, creating a Pi depletion zone in the rhizosphere. To meet plant Pi demand (mean P content approx. 2000 ppm of dry weight), Pi acquisition depends on vigorous root proliferation to accelerate soil exploration, flanked by the chemical and biological modification of the rhizosphere for enhancing Pi accessibility and navigating Pi-associated metal (foremost Al^3+^ and Fe^2+^) toxicities. Different plant Pi acquisition strategies co-evolved with pedogenesis and depend on soil age. In relatively Pi-rich young soils, plants do not necessarily rely on mycorrhizal symbioses and typically remodel root system architecture to forage Pi in shallow soil strata, where the nutrient tends to accumulate in organic litter, followed by microbial re-mineralization (Pi foraging strategy). However, mycorrhizal associations are the most common strategy to scavenge Pi via hyphal outgrowth beyond the root depletion zone from larger volumes of Pi-limited soils (Pi scavenging strategy). Because mycorrhizae are less effective in severely Pi-impoverished or strongly Pi-sorbing soils, formation of cluster roots, characterized by segments of dense lateral rootlets as the sites of profuse carboxylate exudation into small soil volumes (e.g. citrate, malate or oxalate), provides an alternative strategy to extract Pi traces via formidable anion ligand exchange (Pi mining strategy). Thus, non-mycorrhizal plants are typically found at both extremes of the soil fertility spectrum; the ‘Brassicaceae type’ is often associated with rocky Pi-rich landscapes, whereas the ‘Proteaceae type’ is found in severely Pi-impoverished habitats [107–110].

In most dicotyledonous plants, including the non-mycorrhizal reference species *Arabidopsis thaliana*, Pi limitation stimulates formation of a shallow allorhizic root system and expansion of root surface area by attenuating primary root growth, promoting lateral root branching and increasing root hair length and density [111–113]. In Pi-impoverished soils (<15 ppm), the facultative beneficial fungal endophyte, *Colletotrichum tofieldiae*, may colonize *Arabidopsis* roots for increasing Pi acquisition, which likely compensates for the absence of mycorrhizal symbioses in the Brassicaceae [114]. To expedite Pi acquisition, coordinated biochemical processes release metal-chelating ligands (e.g. malate or citrate) and phosphohydrolases into the rhizosphere to mobilize Pi for efficient uptake from mineral and organic sources, respectively [112].

### (b) Plant Pi uptake, storage and distribution

Multiple transport systems facilitate active Pi uptake from soil to root, systemic Pi distribution to organs and tissues and Pi allocation to cells and subcellular compartments. The phosphate transporters (PHT) family has been well-studied in the green lineage. In land plants, members of the five PHT subfamilies localize to the plasma membrane for catalyzing extracellular Pi acquisition (PHT1), to intracellular organelles for meeting metabolic Pi demand in plastids (PHT2 and PHT4), mitochondria (PHT3) and Golgi bodies (PHT4), and to the vacuoles for preventing detrimental accumulation of excess Pi in the cytosol (PHT5) [115,116]. Proteins of the PHT1 subfamily and members of its monophyletic PTA sister clade in green algae (related to yeast Pho84) mediate high-affinity Pi uptake coupled to electrogenic proton symport (2–4 H^+^ per H_2_PO_4_^−^), which requires proton extrusion by plasma membrane H^+^-ATPases. Because proton gradients are difficult to maintain in alkaline moist or aquatic habitats, the PTB group of the PHT family in chlorophytes, which is evolutionary related to bacterial Pit and yeast Pho89 Pi influx transporters (see §4), couples high-affinity Pi symport to sodium ion gradients generated by plasma membrane Na^+^-ATPases. Interestingly, both types of Pi co-transporters co-exist in streptophyte algae and bryophytes. Phylogenetic studies point to a gradual transition during plant terrestrialization from mixed Pi uptake systems (Pi/H^+^ and Pi/Na^+^ symporters) in early diverging land plants to the exclusive dominance of Pi/H^+^ symporters in angiosperms, which adapted to drier habitats [117–119] (figure 1*c*).

In *Arabidopsis*, most of the nine PHT1 proteins are upregulated in Pi-deficient conditions and facilitate Pi/H^+^ symport against a steep concentration gradient (ca. 10 000-fold between soil solution and cytoplasm), with a range of high to medium affinities (*K*_*m*_< 10−100 µM Pi). Genetic analyses revealed PHT1;1−PHT1;4 as the major Pi influx transporters, which are expressed to different degrees in root tissues such as differentiated epidermal and cortex cells, root hairs and root caps. Among those, PHT1;1 and PHT1;4 play an important role in the columella of root tips on Pi-deprived root systems as the hotspots for Pi capture and uptake, which may account for about 20% of the total Pi uptake by seedlings [120–122]. Likewise, root hair development is directly linked to the efficacy of biochemical Pi deficiency responses (e.g. secretion of carboxylates and acid phosphatase activity) and to PHT1-dependent Pi uptake [123].

Land colonization also impacted Pi homeostasis at the level of intracellular Pi storage. Plant vacuoles sequester excess Pi, which may interfere with metabolic reaction rates, and are crucial reservoirs for maintaining a minimum threshold of cytosolic Pi via controlled vacuolar release. However, the form of P stored in vacuoles differs in the green lineage. While the vacuoles of embryophytes may contain up to 75% of total cellular P as free Pi, chlorophytes transiently store excess P as polyP granules complexed with various metal cations in acidocalcisome-like vacuoles [124]. As in yeast, green algae such as *Chlamydomonas* synthesize, store and mobilize vacuolar polyP via the activities of SPX–VTC and SPX–SLC proteins. The transition from polyP to Pi vacuolar storage likely occurred in the early lineage of streptophyte algae (approx. 1.2 Ga), correlating with the loss of SPX–VTC and SPX–SLC families and the rise of tonoplast-located PHT5/VPT vacuolar Pi importers featuring an SPX and Major Facilitator Superfamily (MFS) domain [75]. The transition also correlates with the evolutionary recruitment of plasma membrane-localized GlpT proteins (glycerol-3-phosphate/Pi antiporters, no SPX domain) to the tonoplast for mediating vacuolar Pi efflux (GlpT/VPE proteins) in a common ancestor of embryophytes after the divergence of chlorophytes and streptophytes [75,125]. Polymerization of Pi represents an ancient strategy for P storage and thermodynamic P activation, which was lost in freshwater algae and is most likely absent in land plants [124]. Unlike polyP turnover, direct Pi storage in vacuoles is kinetically swift and more energy-efficient and may have been one critical adaptation to plant terrestrialization (figure 1*c*).

After uptake from the rhizosphere, Pi moves symplastically through several concentric root tissue layers to the central stele, where it is loaded into the apoplastic xylem vessels for transfer to the shoot and distribution within areal organs. The translocation step is mediated by PHO1, a Pi efflux transporter and the prototypical member of the SPX and EXS (ERD1/XPR1/SYG1) domain-containing protein family [126]. *Arabidopsis* PHO1 is primarily expressed in the root xylem parenchyma, and its EXS domain is essential for PHO1 localization to the Golgi and *trans*-Golgi network [127]. Pi export and unloading into the apoplastic space is likely controlled via exocytosis followed by rapid endosomal recycling of PHO1 from the plasma membrane [128]. Members of the PHO1 family in *A. thaliana* and rice (*Oryza sativa*) largely function in vascular tissues and facilitate, for example, Pi allocation to developing seeds [129–131].

### (c) Cellular and systemic regulation of Pi homeostasis

Like in yeast (see §4b; [55]) or humans [132], low abundant (<5 µM) signal molecules of cellular Pi and energy status, i.e. inositol pyrophosphates such as 1,5-IP_8_ [133,134], and their SPX receptor domains play a central role for regulating Pi homeostasis in plants, which has been best studied in *Arabidopsis* and rice [56]. The formation of 1,5-IP_8_ proceeds by the sequential action of ITPK1 (inositol phosphate kinase 1) converting IP_6_ to 5-IP_7_ and VIH1/VIH2 (Vip1 homologs) kinases that are bifunctional enzymes featuring an N-terminal 5-IP_7_ kinase domain and an allosterically communicating C-terminal 1,5-IP_8_ phosphatase domain catalyzing the opposite reaction. High cellular Pi and ATP levels promote VIH kinase whereas cellular Pi shortage reciprocally favours VIH phosphatase activity [65,135]. Thus, energy metabolites oscillate in synchrony with derivative signal molecules to adjust and coordinate metabolism to Pi supply (INPHORS pathway [55]).

SPX sensor domains are present in four plant protein families, which feature a single SPX domain either exclusively (referred to as ‘stand-alone’ SPX polypeptides such as SPX1–SPX4 in *Arabidopsis*) or within the N-terminal region followed by additional domains, termed SPX–MFS proteins (PHT5/VPT family), SPX–EXS proteins (PHO1 family) and SPX– RING (really interesting new gene) proteins (NLA family). SPX1-type polypeptides are potent nuclear repressors of constitutively expressed phosphate starvation response 1 (PHR1) and paralogous PHL (PHR1-like) transcription factors of the plant-specific MYB-CC class. PHR1/PHL and their orthologs in chlorophytes (e.g. PSR1 in *Chlamydomonas*) are key transcriptional regulators of the systemic Pi starvation response in the green lineage [136]. In Pi sufficiency, SPX polypeptides bind to 1,5-IP_8_ with high affinity (7–50 µM [61]), which triggers docking of the SPX:1,5-IP_8_ complex to the dimerized CC domain of PHR1/PHL and, after dimer disruption, interaction with the MYB-like DNA-binding domain, thereby abolishing target gene activation [137,138]. Under Pi-deficient conditions, decreasing 1,5-IP_8_ level attenuates SPX:1,5-IP_8_ complex formation and protein interaction, which promotes PHR1/PHL (hetero)oligomerization and activation of PSI genes via recognition of PHR1-binding sequences (P1BS) promoter elements. Protein degradation of ligand-free SPX and P1BS-mediated SPX gene activation provide positive and negative feedback loops, respectively (figure 2*c*).

Ubiquitination-facilitated proteolysis plays an important role in maintaining Pi homeostasis beyond SPX polypeptide function [139]. For example, under Pi deficiency, the plant-specific protein phosphate transporter traffic facilitator 1 (PHF1), an endoplasmic reticulum (ER) exit factor related to yeast Sec12, mediates the subcellular localization of transcriptionally induced PHT1 Pi influx transporters to the plasma membrane. To prevent excessive Pi uptake in nutrient sufficiency, PHT1 function is post-translationally repressed by interrupting PHT1 localization to the plasma membrane and enhancing PHT1 protein degradation. These processes involve independent and synergistic ubiquitination by PHO2/UBC24, an E2 ubiquitin-conjugating enzyme, and nitrogen limitation adaptation (NLA), an SPX–RING-type E3 ligase. The SPX domain of NLA is thought to interact with PHT1, which promotes PHT1 ubiquitination, endocytosis and multi-vesicular body (MVB)-mediated vacuolar proteolysis. PHO2 reduces PHT1 abundance at the plasma membrane by promoting PHF1 degradation as well as NLA-dependent ubiquitination of PHT1, followed by its 26S proteasome-dependent degradation. Surplus Pi is concurrently sequestered into vacuoles by PHT5/VPT Pi transporters (SPX–MFS), and PHO1-facilitated Pi export to xylem vessels is adjusted by PHO2-initiated PHO1 (SPX–EXS) transfer (via SPX recognition) to MVB-mediated vacuolar proteolysis. Degradation of transcription factors controlling PHO1 expression through the 26S proteasome fine-tune PHO1 function [56,139]. Given the elaborate involvement of SPX sensor domain-containing proteins in the control of Pi homeostasis, in the future it will be important to precisely decipher the molecular network of SPX:inositol pyrophosphate interactions and the metabolic consequences.

A coordinated systemic response to Pi limitation requires the integration of root-to-shoot communication, of different developmental stages and of metabolic crosstalk. In addition to various hormones and nutritional signals, PHR1-dependent expression of mobile non-coding RNAs, which can act as long-distance signals, adds another regulatory layer for the control of cellular and organismic Pi homeostasis [56,140]. For example, Pi deprivation induces evolutionary conserved micro RNAs in the shoot, which are transported through the phloem into the root system to guide the degradation of mRNAs encoding PHO_2_ (miR399) or NLA and PHT5/VPT (miR827). These processes lead to post-transcriptional repression of ubiquitin-mediated protein degradation pathways and enforce transcriptional activation of Pi uptake and translocation in Pi-limiting conditions. PHR1 further induces the expression of long non-coding RNAs that sequester and disable miRNAs in a negative feedback loop known as target mimicry [56].

Genome-wide transcriptome profiling revealed that about two-thirds of the genes responding to Pi shortage (>4000) are controlled by PHR1/PHL master regulators in concert with numerous accessory transcriptional co-factors, which is well reflected by the large and differential epigenetic changes in chromatin accessibility [141,142]. The encoded proteins and, if applicable, their metabolic reactants execute most, if not all, systemic responses related to the maintenance of cellular and organismal Pi homeostasis by means of enhanced Pi acquisition and distribution, enforced Pi recycling, reprioritized Pi allocation and adjustment of general metabolism including the attenuation of photosynthesis [56,143]. Noteworthy is the PHR1/PHL-dependent repression of defence-related genes to restructure the root microbiome, consistent with the idea that plants prioritize Pi nutrition over defence [144]. Likewise, in plant species supporting arbuscular mycorrhizal associations such as rice or *Medicago truncatula*, PHR1/PHL transcription factors and SPX polypeptides assume a central role for initiating and establishing the fungal colonization process essentially at all stages in conditions of systemic Pi deficiency, which emphasizes the importance of plant–AMF interactions for Pi nutrition and homeostasis [145–147].

### (d) Local monitoring of external Pi availability

The heterogenous distribution of not readily accessible Pi in soil forces plants to actively seek the vital nutrient by adjusting root system architecture and by modifying rhizosphere chemistry [111,112,148]. Pi is the predominant mineral nutrient controlling primary root growth and acts as a short-range signal perceived at root tips to guide root development for maximal Pi interception [149]. In *Arabidopsis* roots, insufficient external Pi rapidly attenuates cell elongation (<2 h) and progressively inhibits cell division (<2 days), ultimately corrupting the stem cell niche followed by meristem loss and root growth arrest. These processes are profoundly modified by external Fe availability, pointing to antagonistic Fe–Pi interactions in local Pi sensing [150–156]. While systemic responses to Pi deficiency are largely controlled by the PHR:SPX module, local root responses are primarily governed by two different modules and are only marginally dependent on PHR1.

The first module, the cell-wall-targeted ferroxidase low phosphate root 1 (LPR1) and the single P5-type ATPase phosphate deficiency response 2 (PDR2), which likely function in ER protein quality control, specifically adjusts root cell elongation and division to Pi availability. The expression domains of both proteins overlap in the transition and meristematic zones of root tips. When facing Pi shortage, the LPR1:PDR2 module mediates cell-type-specific Fe^3+^ accumulation in the apoplast, which correlates with reactive oxygen species (ROS) production, and with cell wall modifications including callose deposition. While ROS promotes cell wall stiffening in the transition zone, callose deposition interferes with symplastic communication in the stem cell niche, which blocks movement of the mobile short-root transcription factor, an important regulator of stem cell identity and meristem maintenance [150–152,157,158]. LPR1-dependent root meristem differentiation is thought to involve CLAVATA3/ESR-related 14 (CLE14) peptide signalling [159] and feedback control by Fe-modulated brassinosteroid signalling [160]. Biochemical studies characterized LPR1 as a specific, high-affinity (*K*_*m*_ approx. 2 µM Fe^2+^) ferroxidase, which monitors antagonistic Fe–Pi interactions and detects subtle differences in Fe^2+^ substrate availability as a Pi-dependent cue to adjust root meristem activity via processes most likely initiated by Fe redox cycling. On the other hand, PDR2 counteracts LPR-dependent Fe homeostasis by yet unknown processes [158].

A second module promotes apoplastic Fe^3+^ accumulation and callose deposition preferentially in the transition zone and uncouples the inhibition of cell elongation and cell division in Pi-starved root tips [151,153]. Pi deprivation promotes a nuclear abundance of sensitive to proton rhizotoxicity 1 (STOP1), a master transcription factor regulating stress tolerance to acidity and Al^3+^ toxicity [161], primarily by inhibition of STOP1 ubiquitination and degradation via the 26S proteasome [162,163]. Aluminium-activated malate transporter 1 (ALMT1), a plasma membrane malate channel encoded by a direct STOP1 target gene, facilitates malate efflux into the apoplast of internal cell layers of the root apex. Unlike in the rhizosphere, where malate exudation mobilizes Pi by Al^3+^ or Fe^3+^ chelation for subsequent Pi uptake, malate release into the apoplast and formation of malate–Fe^3+^ complexes is thought to accelerate LPR1-dependent Fe redox cycling, ROS generation and cell wall modifications [151,158]. The tonoplast-localized, bipartite ABC transporter of undefined specificity, aluminium sensitive 3 (ALS3)/sensitive to aluminium rhizotoxicity 1 (STAR1) has been implicated in Al^3+^ tolerance and represses nuclear STOP1 accumulation, possibly by sequestrating trivalent metals or metabolites related to their activity [154,162,164]. As discussed for LPR1, these studies suggest that elevated Fe^3+^ (or Al^3+^) in low Pi serves as a Pi-dependent cue for activating the STOP1:ALMT1 module.

Key components of internal Pi sensing are found in all eukaryotes (e.g. SPX domain) or are present in the green lineage (e.g. MYB-CC domains). However, major players of external Pi sensing seem to have evolved in streptophyte ancestors prior to or upon plant terrestrialization. For example, the absence of LPR1, STOP1, ALMT1, ALS3 or STAR1 orthologs in chlorophytes indicates that PHR1-independent local Pi sensing was a key innovation necessary for successful land colonization [158,165,166]. While the origin of the STOP1:ALMT1 module remains to be studied, horizontal gene transfer (HGT), which played an important role in mastering the challenges during plant terrestrialization, was most likely instrumental in the evolution of LPR1 or ALS3 in embryophytes [15,167,168]. Two major episodes of HGT occurred during the evolution of streptophytes, corresponding to the early evolution of streptophyte algae and the origin of embryophytes, respectively [166]. *Arabidopsis* LPR1 typifies an ancient, hitherto unrecognized ferroxidase cohort that emerged in soil bacteria [158]. Phylogenomics indicates that the common ancestor of streptophytes acquired an LPR1-type ferroxidase from soil bacteria during the first HGT episode, which was probably the case for genes encoding the ALS3/STAR1 transporter complex [158,166]. Likewise, gene orthologs coding for gibberellic acid insensitive (GAI)/repressor of GAI/scarecrow (GRAS) family transcription factors are present in soil bacteria and streptophyte algae [168]. Notably, at least two GRAS family members control stem cell niche identity and maintenance in *Arabidopsis* roots [169], processes inhibited by LPR1-dependent callose deposition in Pi-deprived root tips [150]. Because the development of rhizoids and root hairs are evolutionary conserved [91,92], the putative role of LPR1 in root hair development is particularly intriguing [170]. Thus, the prospect arises that HGT facilitated the evolution of local Pi sensing and Pi acquisition during plant terrestrialization (figure 1*c*).

## 7. Anthropogenic derangement of the global phosphorus cycle

Our planet has entered the Anthropocene epoch, the present era in which humanity significantly impacts Earth system functioning and the geological stratigraphic record. While the potential start date of the new epoch is debated among geologists, the unprecedented changes since the mid-twentieth century are certainly distinctive. The so-called Great Acceleration is marked by the exponential surge in world population growth (surpassed more than twice by farm animal biomass), in energy consumption, in industrial materials output (already exceeding the global living biomass), in agricultural food and feed production or in global economic transportation [6,171–173].

### (a) Human intensification of global Pi mobilization

Human activities, foremost modern large-scale agriculture, have profoundly altered the interconnected global C, N and P cycles [7,174]. In particular, the biogeochemical flow of Pi has been accelerated in unparalleled ways, primarily by the production of mineral fertilizers and animal feed supplements from high-grade P rock resources [175]. Application of inorganic P (together with N and K) fertilizers has immensely increased soil fertility and crop production. However, beyond its intended benefits, global run-off and leaching of excess Pi from fields often lead to detrimental environmental consequences such as eutrophication, which threatens anoxia of freshwater bodies and coastal seas [174]. Inorganic P fertilizer production and Pi input to the terrestrial biosphere exceeds more than twofold the natural Pi weathering rate (e.g. 23 Mt P were used in 2008 to fertilize almost exclusively arable soils [176]), a ratio expected to notably increase with continual world population growth. Similarly, while agricultural soils received an equivalent of 10% of total soil P content by fertilizer input between 1950 and 2000, this fraction sharply increased to 47% by 2017 [5,175]. A second major factor intensifying geochemical Pi flows from continents to oceans is the accelerated soil erosion and run-off due to deforestation, arable land degradation, expansion of agricultural cropping systems and relentless urbanization campaigns (anthropogenic and natural Pi weathering rates in 2008 were about equal at 10 Mt P each [100,176]). Recycling of organic waste excreted by the global populations of humans and their livestock returns about 15 Mt P to soil annually. Thus, since the mid-nineteenth century, when industrial production of Pi fertilizers was launched in the few global regions hosting high-grade P deposits, irreversible (at least over civilizational timescales) Pi mobilization in the biosphere has probably quadrupled when compared to pre-industrial levels [5].

International trade of P fertilizers and agricultural (organic P) products between continents amounts to 12 Mt P annually, which points to another unfavourable aspect of the modern P cycle [173]. Its anthropogenic intensification causes the geographic dispersal of the non-renewable nutrient around the globe. Unlike the closed biospheric N cycle by which reactive forms of the element are biotically recycled to inert atmospheric N_2_ over biological life spans (denitrification), the globally mobilized and thinly distributed Pi requires geological timescales for recycling and concentrating into sediments by abiotic processes. Regardless, feeding the growing world population with its unabated appetite for animal meat, together with global efforts to eliminate undernutrition and malnutrition, will considerably increase the demand for mineral Pi fertilizer input in crop production systems, projected at 27–31 Mt P annually by 2050 (International Fertilizer Association). Just a mere upscaling of past or current unsustainable practices will be in conflict with the harmful impact on ecosystems caused by Pi excess (eutrophication of surface waters) and by environmental hazards of mineral Pi fertilizer production, which will be increasingly reliant on lower-grade P rock reserves contaminated with heavy metals (foremost Cd) and radioactive nuclides (mainly U) [5].

### (b) Conclusion

The formidable challenges ahead will be best addressed by minimizing the initial Pi input necessary for plant-based feed and food production, rather than mitigating its detrimental environmental consequences. Obviously, less meat consumption by humanity will substantially reduce Pi fertilization for growing animal feed (largely cereals and legumes), avert the use of mineral Pi supplements for substituting phytic acid in such fodder (a major P storage form humans or monogastric livestock cannot digest) and thus prevent the large losses of P in excreted animal manure [5]. Because only less than 25% of the Pi applied as fertilizers are bioavailable to field crops, implementation of best agronomic management practices for optimal Pi fertilization, tailored to local and regional production systems, provides a second strategy [177]. Lastly, the largest economic and environmental benefits may be realized by opportunities for plant breeding and by state-of-the-art genetic improvement of plant Pi acquisition and use efficiencies (PAE/PUE), which will support greater yields in Pi-deficient soils or optimal crop production in well-fertilized soils by reducing Pi input or by lowering soil P content, respectively [178,179]. Current efforts for raising the PAE or PUE of cultivated plants, which are complex agronomic traits difficult to manipulate for crop success in the field, centre on various processes highlighted in this review. For example, current research aims at dissecting the genetic and molecular circuits controlling the development of root hairs, root system architecture and fungal symbiotic associations, the chemical and microbial modification of the rhizosphere for Pi mobilization, the uptake and organismic allocation of Pi, the remobilization of Pi from cellular constituents and compartments, the coordination of Pi-centred metabolic crosstalk or, last but not least, at understanding the mechanisms of Pi sensing and long-distance signalling [56,180]. Continual funding of both fundamental and applied research will be acutely necessary for readjusting the global P cycle deranged by anthropogenic activity.

## Data Availability

This article has no additional data.

## Appendix A

See table 1.

**Table 1.** Glossary.

(a) Relevant geological terms
**Apatite:** Most common phosphate minerals, generalized by Ca_5_(PO_4_)_3_(F,Cl,OH); present in the three main rock types (igneous, metamorphic and sedimentary).
**Authigenic minerals:** Formed by *in situ* inorganic precipitation on the sea floor and within the sediment column, as opposed to detrital sediments, which are formed by physically dislocated (e.g. by gravity or wind) eroded rocks and minerals.
**Chronosequence**: A set of ecological sites that differ in age but share similar soil types and environmental (climatic) conditions.
**Clay minerals**: Commonly formed by prolonged chemical weathering of silicate-bearing rocks.
**Felsic rock**: Igneous rock category (e.g. granite); minerals enriched in elements such as Si, O, Al, Na and K (>45% Si).
**Fulgurite rock**: Natural tubes of sintered soil, sand or rock that may form by lightning strikes.
**Igneous rock**: From Latin *igneus* ‘fiery’. The slow cooling and crystallization of magma forms intrusive, coarse-grained rock (e.g. granite); the rapid cooling and solidification of lava forms extrusive, fine-grained rock (e.g. basalt) or natural glass (e.g. obsidian).
**Lithosphere**: The solid outer part of the Earth, including the upper mantel and the crust.
**Lithophilic elements**: Chemical elements that readily combine with oxygen and therefore do not descend into the Earth’s core.
**Mafic rock**: Igneous rock category (e.g. basalt); minerals poor in silica (<45% Si) but enriched in magnesium and iron. Ultramafic rocks are especially poor in silica (<40% Si).
**Metamorphic rock**: Formed by the physical or chemical transformation of pre-existing rock at elevated temperature and pressure.
**Olivine**: Important rock-forming mineral widespread in ultramafic igneous rocks, generalized as (Fe,Mg)_2_SiO_4_, in which PO_4_ may replace SiO_4_.
**Phosphorite**: A phosphate-rich, authigenic sedimentary rock containing apatite (up to 80%).
**Regolith**: Blanket of superficial deposits (dust, gravel and broken rock) covering bedrock.
**Schreibersite**: (Fe,Ni)_3_P, a rare iron–nickel phosphide mineral on Earth, but the most abundant phosphide in the cosmos (a major component of early meteorite flux).
**Sedimentary rock**: Formed by the accumulation of mineral particles (erosion and chemical precipitation) or materials of organic origin (dead organisms and biochemical precipitation), followed by lithification (deposition, compaction and cementation); processes known as diagenesis.
**Serpentinization**: Metamorphic transformation of olivine-rich ultramafic or mafic rocks by hydration and oxidation. The reaction is highly exothermic and may form non-volcanic hydrothermal vents.
**Siderophilic elements**: Chemical elements, particularly transition metals, which tend to migrate to the Earth’s core because they readily dissolve in iron. Phosphorus reacts with metals to form phosphides due to its siderophilic character at high temperatures.
(b) Relevant biological terms and frequent abbreviations
**ABC transporter:** A transport system superfamily characterized by an ATP-binding cassette present in all phyla from prokaryotes to humans. A typical ABC transporter is composed of two nucleotide-binding domains that energize transport via ATP hydrolysis and of two membrane-spanning domains that provide a channel for the transport substrate.
**Acidocalcisome:** Acidic organelles in a diverse range of organisms from bacteria to human cells, which contain a matrix of pyrophosphate and polyphosphates bound with calcium and other cations.
**Allorhizic root system**: Common in dicotyledonous plants, defined by a single primary root, which gives rise to secondary and higher-order lateral roots (also called tap root system). In contrast, homorhizic root systems (or fibrous roots) are found mainly in monocotyledonous plants and are dominated by seminal and adventitious roots.
**Arbuscular mycorrhiza**: A symbiotic interaction between fungi and plants. Arbuscular mycorrhizal fungi (AMF) penetrate the root cortical cells of host plants to form unique tree-like structures, the arbuscules.
**Ectomycorrhiza (EM):** A symbiotic interaction between fungi and plants, usually restricted to woody plants. Unlike arbuscular mycorrhizae, ectomycorrhizal fungi do not penetrate host cell walls. EM fungi form an intercellular interface (Hartig net), consisting of highly branched hyphae forming a dense lattice between epidermal and cortical root cells, and a fungal mantel that envelops the host roots.
**Eutrophication**. Excessive pollution of surface waters with mineral nutrients (e.g. from agricultural run-off), leading to algae blooms and rapid bacterial growth, followed by oxygen depletion (anoxia) and substantial ecological degradation.
**Inositol (pyro)phosphates**: Phosphorylated and pyrophosphorylated derivatives of myo-inositol, e.g. inositol 1,4,5-trisphosphate (IP_3_) or inositol 1,5-bis(diphosphate) 2,3,4,6-tetrakisphosphate (1,5-IP_8_), which are involved in the regulation of several cellular processes via their interaction with specific proteins, such as SPX domain proteins with 1,5-IP_8_.
**Net primary production**: The difference in biomass per unit area and time between the assimilation of carbon dioxide into organic matter and the concomitant loss due to respiration.
**PHO regulon/pathway:** A set of co-regulated PHO (phosphate) genes that dynamically adjust the metabolism and physiology of bacteria and fungi to fluctuating Pi availability.
**Polyphosphate (polyP)**: A linear polymer of 3–1000 orthophosphate units connected via high-energy phosphoric anhydride bonds.
**PSI genes**: Large sets of Phosphate-Starvation-Inducible genes that dynamically adjust the metabolism and physiology of algae and land plants to fluctuating Pi availability.
**Phosphorus starvation response 1 (PSR1)/phosphate starvation response 1 (PHR1)**: Transcriptional master regulators of PSI gene expression in *Chlamydomonas* (PSR1) and *Arabidopsis* (PHR1).
**SPX domain**: Sensor domains (SYG1/PHO81/XR1) of inositol pyrophosphates found in proteins related to Pi homeostasis with functions in signalling, transport, storage and polyP turnover.
**Terrabacteria:** A taxon comprising about two-thirds of prokaryotic species, including Cyanobacteria, the gram-positive phyla (Actinobacteria and Firmicutes), and two additional phyla (Chloroflexi and Deinococcus-Thermus), which evolved important adaptations to environmental hazards on land (e.g. desiccation, UV radiation or high salinity) [181].
